# Structural and biochemical characterization of the KLHL3–WNK kinase interaction important in blood pressure regulation

**DOI:** 10.1042/BJ20140153

**Published:** 2014-05-13

**Authors:** Frances-Rose Schumacher, Fiona J. Sorrell, Dario R. Alessi, Alex N. Bullock, Thimo Kurz

**Affiliations:** *MRC Protein Phosphorylation and Ubiquitylation Unit, College of Life Sciences, University of Dundee, Dow Street, Dundee DD1 5EH, Scotland, U.K.; †Structural Genomics Consortium, University of Oxford, Old Road Campus, Roosevelt Drive, Oxford OX3 7DQ, U.K.

**Keywords:** Bric-a-brac, Tramtrack, and Broad complex (BTB domain), Cullin, hypertension, Kelch-like protein (KLHL), Kelch-like protein 2 (KLHL2), ubiquitin, BTB, Bric-a-brac, Tramtrack, and Broad complex, CRL3^KLHL3^, Cullin3-RING ligase in complex with KLHL, CUL3, Cullin3, KEAP1, Kelch-like enoyl-CoA hydratase-associated protein 1, KLHL, Kelch-like protein, NCC, Na^+^/Cl^−^ ion co-transporter, NKCC2, Na^+^/K^+^/2Cl^−^ co-transporter 2, NRF2, nuclear factor-erythroid 2-related factor 2, OSR1, oxidative stress-responsive kinase 1, rTEV, recombinant tobacco etch virus, RT-PCR, reverse transcription–PCR, SPAK, SPS1-related proline/alanine-rich kinase, TCEP, tris-(2-carboxyethyl)phosphine, TEV, tobacco etch virus, WNK, with no lysine (K)

## Abstract

WNK1 [with no lysine (K)] and WNK4 regulate blood pressure by controlling the activity of ion co-transporters in the kidney. Groundbreaking work has revealed that the ubiquitylation and hence levels of WNK isoforms are controlled by a Cullin-RING E3 ubiquitin ligase complex (CRL3^KLHL3^) that utilizes CUL3 (Cullin3) and its substrate adaptor, KLHL3 (Kelch-like protein 3). Loss-of-function mutations in either CUL3 or KLHL3 cause the hereditary high blood pressure disease Gordon's syndrome by stabilizing WNK isoforms. KLHL3 binds to a highly conserved degron motif located within the C-terminal non-catalytic domain of WNK isoforms. This interaction is essential for ubiquitylation by CRL3^KLHL3^ and disease-causing mutations in WNK4 and KLHL3 exert their effects on blood pressure by disrupting this interaction. In the present study, we report on the crystal structure of the KLHL3 Kelch domain in complex with the WNK4 degron motif. This reveals an intricate web of interactions between conserved residues on the surface of the Kelch domain β-propeller and the WNK4 degron motif. Importantly, many of the disease-causing mutations inhibit binding by disrupting critical interface contacts. We also present the structure of the WNK4 degron motif in complex with KLHL2 that has also been reported to bind WNK4. This confirms that KLHL2 interacts with WNK kinases in a similar manner to KLHL3, but strikingly different to how another KLHL protein, KEAP1 (Kelch-like enoyl-CoA hydratase-associated protein 1), binds to its substrate NRF2 (nuclear factor-erythroid 2-related factor 2). The present study provides further insights into how Kelch-like adaptor proteins recognize their substrates and provides a structural basis for how mutations in WNK4 and KLHL3 lead to hypertension.

## Abstract 2

WNK kinases regulate mammalian blood pressure. The level of WNK protein in a cell is regulated by the KLHL3–CUL3 ubiquitin ligase. We define the interaction between KLHL3 and WNK, identifying the WNK degron, and present the crystal structure of the KLHL3–WNK degron complex.

## INTRODUCTION

The WNK [with no lysine (K)] kinases play central roles in regulating mammalian blood pressure by initiating a signalling pathway that controls the activity of critical ion co-transporters in the kidney NCC (Na^+^/Cl^−^ ion co-transporter) and NKCC2 (Na^+^/K^+^/2Cl^−^ co-transporter 2) [1–3]. There are four widely expressed mammalian WNK isoforms (WNK1, WNK2, WNK3 and WNK4) that exert their physiological effects by activating two highly related protein kinases termed SPAK [SPS1-related proline/alanine-rich kinase; also known as STK39 (serine threonine kinase 39)] and OSR1 (oxidative stress-responsive kinase 1) [1,4–10]. Once activated, SPAK/OSR1 promote salt retention by phosphorylating and activating sodium ion co-transporters (NKCC1, NKCC2 and NCC) [1,4,6–9,11,12] and inhibiting KCCs (potassium ion co-transporters) [1,13].

The importance of the WNK system in controlling blood pressure was first illustrated by the finding that intronic mutations within the human *WNK1* gene that increase *WNK1* mRNA expression, and presumably WNK1 protein expression, result in Gordon's hypertension syndrome (also known as pseudohypoaldosteronism type II) [3,10,14]. Mutations within a non-catalytic highly acidic motif of WNK4 located C-terminal to the kinase domain (residues 557–567) also cause Gordon's syndrome [10,15]. How mutations within this region, which is conserved in all WNK isoforms, stimulated the WNK signalling system was not clear. However, findings from previous studies suggest that this acidic region plays a critical role in controlling the ubiquitylation, and hence stability of WNK4, by operating as a degron motif for the CRL3^KLHL3^ E3 ligase complex, which includes CUL3 (Cullin3) and the substrate adaptor KLHL3 (Kelch-like protein 3) [4–9]. The current data point towards a model in which the CRL3^KLHL3^ complex binds to the acidic degron motif leading to WNK4 ubiquitylation and proteasomal degradation. This conclusion is supported by the finding that mutations in KLHL3 that inhibit binding to either CUL3 or WNK4 also cause Gordon's hypertension syndrome in humans [1,4,6–9,11,12]. Moreover, *in vitro*, the CRL3^KLHL3^ complex efficiently ubiquitylates WNK1 and WNK4 in a manner that is prevented by the most commonly described KLHL3 mutation (R528H) [6]. Consistent with the acidic region operating as a degron motif, recent work has revealed that a WNK4 knock-in mouse displaying a Gordon's syndrome mutation within this motif (WNK4 [D561A]) possesses markedly elevated levels of WNK4 in the kidney [7]. Furthermore, the D561A mutation decreased WNK4 ubiquitylation by CRL3^KLHL3^ in an overexpression system [7]. These studies emphasize the importance that the interaction between the KLHL3 and acidic degron motif play in controlling the ubiquitylation and stability of WNK isoforms and therefore blood pressure.

CUL3 acts as a scaffolding protein that, through its N-terminus, binds to the BTB (Bric-a-brac, Tramtrack, and Broad complex) domain in KLHL3 [16]. KLHL3 is the substrate-recognition subunit of the ubiquitin ligase complex, recruiting substrates for ubiquitylation via its associated Kelch domain [17–19]. The CUL3 C-terminus also interacts with the RING-finger protein RBX1 (ring-box 1), which binds to ubiquitin-charged E2 enzymes and promotes transfer of ubiquitin on to the bound substrate [20]. In addition to KLHL3, CUL3 can form distinct E3 ligase complexes with other BTB-domain-containing proteins [21,22]. There are close to 200 BTB domain proteins encoded in the human genome, and although thus far only 11 BTB adaptor subunits have been well characterized in humans, there are likely to be numerous more CUL3 adaptor complexes that interact with many diverse substrates [19]. For example, CUL3 interacts with the BTB-Kelch-like protein KEAP1 (Kelch-like enoyl-CoA hydratase-associated protein 1), which binds to and ubiquitylates the transcription factor NRF2 (nuclear factor-erythroid 2-related factor 2) [23]. Dimerization of the BTB domain affords two Kelch domains per CUL3 adaptor. In this instance, NRF2 possesses one high affinity degron and a second lower-affinity degron-binding site. Both sites need to be engaged by two Kelch domains of dimeric KEAP1 to appropriately orient NRF2 for ubiquitylation [24]. Another member of the BTB-Kelch family of proteins is KLHL2 (also known as Mayven). KLHL2 shares 84% sequence identity with KLHL3 across the Kelch domain, and 86% identity overall (see Figure 2A). KLHL2 has also been implicated recently as a CUL3 substrate adaptor for WNK kinases [25].

In the present study, we scrutinize the interaction between the WNK degron motif and KLHL3 as well as KLHL2. The data reveal the fundamental features of this interaction and explain how several of the disease-causing mutations disrupt binding. Our results also reveal that the WNK degron motif binds to KLHL3 in a markedly different manner to how NRF2 binds to KEAP1, which is the only other BTB-Kelch protein for which substrate interactions have been analysed at the structural level thus far.

## MATERIALS AND METHODS

### Materials

Lumio Green, Colloidal Blue staining kit and precast SDS polyacrylamide BisTris gels were from Invitrogen. All peptides were synthesized by GL Biochem to a purity of >95%; peptide sequences were determined by MS.

### Plasmids

The full-length coding region of KLHL3 (NM_017415.2) was amplified from skeletal muscle total RNA (Agilent) by using RT-PCR (reverse transcription–PCR) according to the manufacturer's protocol (SuperScript® III One Step RT-PCR, Invitrogen). Fragments of KLHL3 comprising residues 290–587 and 296–587 were subsequently PCR-amplified adding flanking BglII and NotI restriction and shuttled directly into the bacterial expression vector pGEX6P-1 downstream of GST and a PreScission Protease cleavage site. Point mutations were introduced using the QuikChange® method (Stratagene) in conjunction with KOD Hot Start DNA polymerase (Novagen). KEAP1 (NM_203500.1) was amplified from EST IMAGE 3163902 and subcloned as a BamHI/NotI insert into a modified pFastbacDualbaculoviral vector containing an N-terminal DAC tag [26] with a TEV (tobacco etch virus) protease site downstream of the DAC tag. DNA sequencing was performed by The Sequencing Service, College of Life Sciences, University of Dundee, U.K. (www.dnaseq.co.uk).

For structure determination, human KLHL2 (IMAGE clone 4791972; residues 294–593) and human KLHL3 (NM_017415.2; residues 298–587) were cloned into the expression vector pNIC28-Bsa4 (GenBank® accession number EF198106) by ligation-independent cloning. This vector encodes for an N-terminal His_6_ tag and rTEV (recombinant TEV) cleavage site.

### General methods

Restriction enzyme digests, DNA ligations and other DNA procedures, were performed using standard protocols. Site-specific mutagenesis was achieved using the standard QuikChange® method (Stratagene).

### Protein purification for fluorescent polarization

All pGEX-6P-1 constructs were transformed into BL21 *Escherichia coli* cells, and 2 litre cultures were grown at 37°C in LB broth containing 100 μg/ml ampicillin until the *D*_600_ reached 0.6. IPTG was then added (50 μM) and the cells were cultured for a further 16 h at 16°C. Cells were isolated by centrifugation, resuspended in ice-cold lysis buffer and lysed by sonication. The lysates were centrifuged at 4°C for 15 min at 26000 ***g***. The GST-fusion proteins were affinity purified on 0.5 ml of glutathione–Sepharose and eluted in buffer containing 20 mM glutathione.

KLHL3 full-length protein (residues 1–587) was expressed and purified as a DAC-(TEV protease)KLHL3 fusion protein in Sf21 cells as described previously in [6]. KEAP1 full-length protein (residues 1–624) was expressed as a DAC-(TEV protease)-KEAP1 fusion protein in insect Sf21 cells [26]. The fusion protein was purified by capture on ampicillin–Sepharose followed by treatment with TEV protease to release KEAP1 and further purification was achieved by size-exclusion chromatography (S75 Superose; GE Healthcare).

### Fluorescence polarization

Fluorescence polarization measurements were performed at 25°C with purified KLHL3 proteins in 50 mM Tris/HCl, pH 7.5, 150 mM NaCl and 2 mM DTT. The concentration of the KLHL3 proteins, KLHL3, GST–KLHL3^290–587^, KEAP1 and GST–KLHL2^296–593^, was determined by measuring their absorbance at 280 nm and calculated using the molar absorption coefficient determined by the ProtParam Online tool [27]. All peptides contained an N-terminal linker required for conjugating to the Lumio Green fluorophore (CCPGCCGGGG) and were initially resuspended in 50 mM ammonium biocarbonate, pH 8. Peptide labelling was achieved by incubating 10 nM of each peptide in a 0.5 ml reaction mixture of 20 μM Lumio Green in 50 mM Tris/HCl, pH 7.5, 150 mM NaCl and 2 mM DTT. Reactions were left to proceed in the dark for 2 h. The peptides were then dialysed for 16 h into 50 mM Tris/HCl, pH 7.5, 150 mM NaCl and 2 mM DTT using a Micro DispoDIALYZER with a 100 Da molecular-mass cut-off (Harvard Apparatus). For fluorescence polarization, mixtures were set up containing the indicated concentration of protein, 10 nM Lumio-Green-labelled peptide in a final volume of 30 μl. All individual bindings were performed in duplicate with at least 12 data points per curve. Fluorescence polarization measures were made using a BMG PheraStar plate reader, with an excitation wavelength of 485 nm and an emission wavelength of 538 nm, and measurements were corrected to the fluorescent probe alone. Data analysis and graphing were then performed in GraphPad Prism6; One Site Specific binding with Hill slope was assumed (model *Y*=*B*_max_×*X*^h^/*K*_d_^h^+*X*^h^) and the disassociation constant and associated S.E.M. was obtained. All experimental bindings were repeated a minimum of two times and comparable results to those shown in the present study were obtained.

### Protein expression and purification for crystallization

For structure determination, human KLHL2 (residues 294–593) and KLHL3 (residues 298–587) were expressed from the vector pNIC28-Bsa4 in BL21(DE3)-R3-pRARE cells (a phage-resistant derivative of Rosetta2, Novagen). Cultures (1 litre) in Terrific broth were incubated at 37°C until *D*_600_ reached 2.0 and then cooled to 18°C and supplemented with 0.5 mM IPTG to induce protein expression overnight. Cells were harvested by centrifugation, resuspended in binding buffer {50 mM Hepes, pH 7.5, 500 mM NaCl, 5 mM imidazole, 5% glycerol and 0.5 mM TCEP [tris-(2-carboxyethyl)phosphine]} and lysed by sonication. The His_6_-tagged proteins were purified using Ni^2+^–Sepharose resin (GE Healthcare) and eluted stepwise in binding buffer with 100–250 mM imidazole. Removal of the His_6_ tag was performed at 4°C overnight using rTEV protease. Proteins were then further purified by anion exchange chromatography using a 5 ml HiTrap SP (KLHL2) or a 5 ml HiTrap Q (KLHL3) column (GE Healthcare), followed by buffer-exchange into 50 mM Hepes, pH 7.5, 300 mM NaCl, 5% glycerol and 0.5 mM TCEP using size-exclusion chromatography (Superdex 200 16/60, GE Healthcare).

### Crystallization and data collection

The purified proteins were concentrated to 9 mg/ml using a 10 kDa molecular-mass cut-off centrifugal concentrator (Millipore). The 11-residue WNK4 peptide (EPEEPEADQHQ) was added to a final concentration of 2 mM. The protein–peptide solution was incubated on ice for approximately 30 min before preparation of sitting-drop vapour-diffusion crystallization plates. Crystals grew under multiple conditions using either freshly prepared or frozen protein. The best-diffracting crystals of the KLHL2 complex were obtained at 20°C by mixing 100 nl of protein with 50 nl of a reservoir solution containing 0.1 M Hepes, pH 7.2, 2.5 M ammonium sulfate and 2% PEG 400. Crystals of the KLHL3 complex were grown similarly using 0.1 M acetate, pH 4.3, 0.2 M ammonium sulfate and 25–35% PEG 4000. After 3 h of incubation, the drops were spiked with 20 nl of seed-stock solution. Seed stock was prepared from poorly formed crystals of KLHL2 or KLHL3 grown during previous rounds of crystal optimization, which were transferred into an Eppendorf containing 50–100 μl of reservoir solution and a seed bead (Hampton Research), then vortex-mixed for 2 min. Before vitrification in liquid nitrogen, crystals were cryoprotected by direct addition of reservoir solution supplemented with 25% ethylene glycol. Diffraction data were collected on beamline I02 at Diamond Light Source, Didcot, U.K.

### Structure determination

Diffraction data for both the KLHL2 and KLHL3 crystals were integrated using Mosflm [28] and scaled using SCALA [29] from the CCP4 software suite [30]. Molecular replacement was performed with Phaser MR in CCP4 using PDB code 2XN4 chain A (apo Kelch domain of KLHL2) as the search model. COOT [31] was used for manual model building and refinement, whereas REFMAC [32] and PHENIX.REFINE [33] were used for automated refinement. TLS (Translation–Libration–Screw-rotation) parameters were included at later stages of refinement. Tools in COOT, PHENIX and MolProbity [34] were used to validate the structures. Structure factors and co-ordinates have been deposited in the PDB under 4CHB (KLHL2) and 4CH9 (KLHL3).

## RESULTS

### Defining the KLHL3-binding residues within the WNK4 acidic degron motif

**Figure 1:**
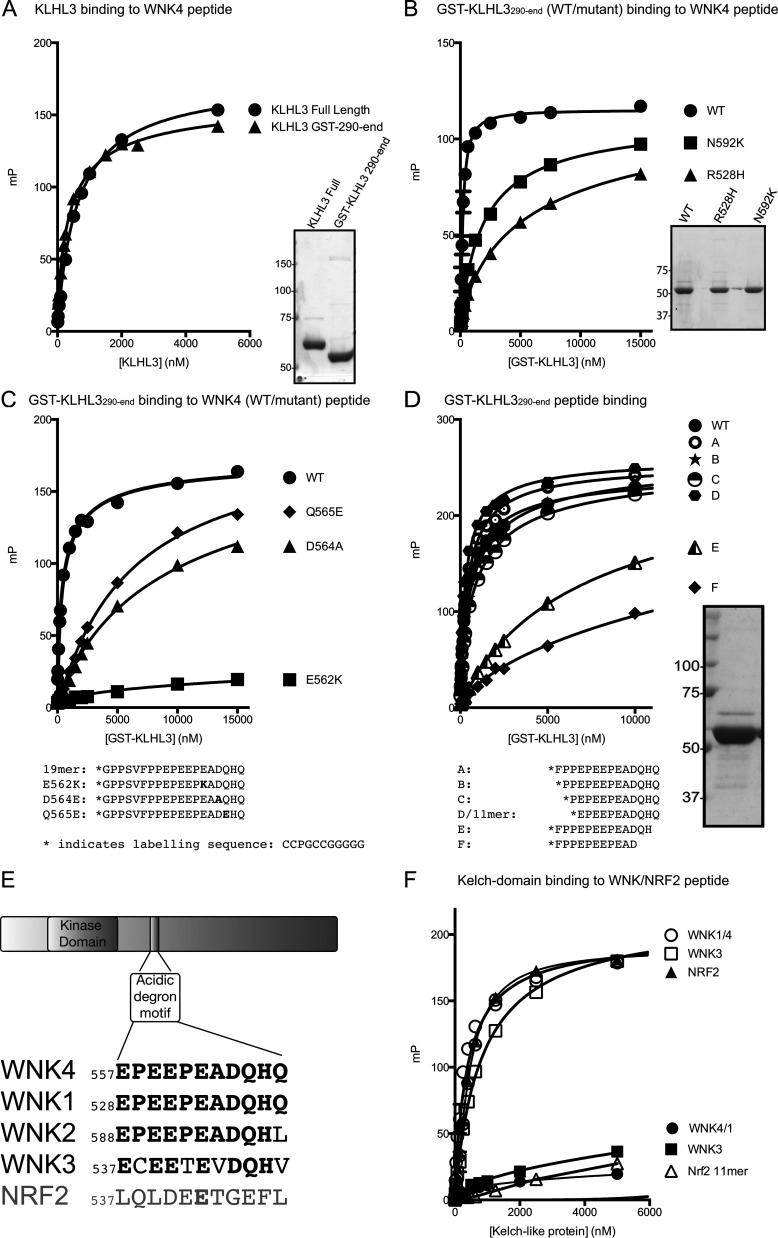
Analysis of KLHL3–WNK interaction by fluorescence polarization (**A**–**D** and **F**) Purified KLHL3 was diluted appropriately and mixed at a 1:1 volume ratio with 20 nM Lumino-Green-labelled WNK peptide to the concentration stated in the Figure, with the peptide concentration consistent at 10 nM, and fluorescent polarization measurements were made. Binding curves, assuming one-site-specific binding, were then generated with Prism6 using milli-polarization (mP) units. The determined dissociation constant for each binding experiment is shown in Table 1. The purified KLHL3 proteins used in the binding experiments were analysed on SDS/PAGE and are shown in panels to the right of the binding curve. Molecular masses are indicated on the left of the gels. (**A**) Comparison of full-length KLHL3 and the isolated Kelch-domain of KLHL3 binding to the 19-residue WNK4 degron peptide (GPPSVFPPEPEEPEADQHQ, residues 549–567). (**B**) Influence of KLHL3 Gordon's syndrome mutations on WNK4 peptide binding. Purified GST-KLHL3^290–587^ wild-type, or the point mutants, GST–KLHL3^290–587^ R528H, GST–KLHL3^290–587^ N259K, were analysed for their ability to bind the 19-residue WNK4 degron peptide. (**C**) Effect of WNK4 Gordon's syndrome mutations on KLHL3 binding. Purified GST–KLHL3^290–587^ wild-type protein was mixed with the 19-residue wild-type (WT) or indicated point mutants (E562K, D564A and Q565E) of the WNK4 degron peptide. The sequences of all peptides used are shown in the bottom panel exclude the N-terminal sequence (CCPGCCGGGG) required for Lumio Green labelling. (**D**) Impact of shortening the WNK4 peptide on KLHL3 binding. The ability of the indicated WNK4 peptides A–F (the sequences of which are shown below the binding curve) to bind purified GST–KLHL3^290–587^ protein was evaluated. (**E**) Location and conservation of degron motif in WNK isoforms. The upper panel displays the domain organisation of WNK isoforms and the location of the degron motif within the C-terminal non-catalytic domain. The lower panel displays an alignment of the degron motif in different WNK isoforms with that of the previously characterized degron motif of the NRF2 transcription factor that interacts with the KEAP1 Kelch-like protein [38]. Identical residues are shaded in black. (**F**) Evaluation of the ability of WNK1/4, WNK3 and NRF2 degron peptides to interact with GST–KLHL3^290–587^ (open symbols) and full-length KEAP1 (closed symbols). WT, wild-type.

As outlined in the Introduction, previous work highlighted that residues within the WNK4 acidic degron motif play a critical role in binding to KLHL3 [4,6–9]. To confirm these data and to set up a quantitative binding assay to monitor the interaction of KLHL3 with the motif, we expressed and purified either full-length KLHL3 or the isolated C-terminal Kelch domain (residues 290–587) and used fluorescence polarization to study the interaction of a 19-residue peptide encompassing the conserved acidic degron of WNK4 (residues 549–567: GPPSVFPPEPEEPEADQHQ). This revealed that full-length KLHL3, as well as the isolated KLHL3 Kelch domain, bound to the 19-residue WNK4 peptide with high affinity (*K*_d_=0.3–0.9 μM) (Figure 1A and Table 1). Replacement of KLHL3 Gordon's syndrome-causing mutations located within the Kelch domain (R528H or N529K) reduced binding to the WNK4 degron peptide approximately 10-fold (Figure 1B and Table 1). Introduction of D564A or Q565E disease-causing mutations within the WNK4 degron similarly resulted in a 10-fold reduction in binding, whereas the E562K mutation virtually abolished binding (Figure 1C and Table 1).

**Table 1:** Disassociation constants (*K*_d_) for peptides binding to different Kelch-like proteins *K*_d_ values calculated from the different fluorescence polarization binding studies shown in Figures 1 and 2. The Kelch-domain protein construct, peptide sequence and calculated *K*_d_ values for each experiment are indicated. All peptide sequences shown exclude the N-terminal sequence (CCPGCCGGGG) that was required for Lumio Green labelling and was present on all peptides listed in this Table. One site-specific binding with Hill slope was assumed and the disassociation constant (*K*_d_), determined in Prism6 using the curves generated and shown in the associated Figures, is noted, along with the S.E.M. Peptides for which binding was too low to measure accurately (*K*_d_>50 μM) are marked LB (low binding). WT, wild-type.

Figure	Kelch protein	Peptide sequence	*K*_d_ (μM)
1(A)	KLHL3 full-length	GPPSVFPPEPEEPEADQHQ (WNK4 WT 549–567)	0.62±0.04
	GST–KLHL3^290–587^	GPPSVFPPEPEEPEADQHQ (WNK4 WT 549–567)	0.38±0.03
1(B)	GST–KLHL3^290–587^	GPPSVFPPEPEEPEADQHQ (WNK4 WT 549–567)	0.19±0.01
	GST–KLHL3^290–587^R528H	GPPSVFPPEPEEPEADQHQ (WNK4 WT 549–567)	1.93±0.15
	GST–KLHL3^290–587^N529K	GPPSVFPPEPEEPEADQHQ (WNK4 WT 549–567)	6.60±1.02
1(C)	GST–KLHL3^290–587^	GPPSVFPPEPEEPEADQHQ (WNK4 WT 549–567)	0.48±0.03
	GST–KLHL3^290–587^	GPPSVFPPEPEEPKADQHQ (WNK4 E562K 549–567)	LB
	GST–KLHL3^290–587^	GPPSVFPPEPEEPEAAQHQ (WNK4 D564A 549–567)	LB
	GST–KLHL3^290–587^	GPPSVFPPEPEEPEADEHQ (WNK4 Q565E 549–567)	6.21±1.02
1(D)	GST–KLHL3^290–587^	GPPSVFPPEPEEPEADQHQ (WNK4 WT 549–567)	0.90±0.06
	GST–KLHL3^290–587^	FPPEPEEPEADQHQ (WNK4 WT 554–567)	0.71±0.05
	GST–KLHL3^290–587^	PPEPEEPEADQHQ (WNK4 WT 555–567)	0.91±0.06
	GST–KLHL3^290–587^	PEPEEPEADQHQ (WNK4 WT 556–567)	0.29±0.05
	GST–KLHL3^290–587^	EPEEPEADQHQ (WNK4 WT 557–567)	0.69±0.04
	GST–KLHL3^290–587^	FPPEPEEPEADQH (WNK4 WT 554–566)	7.81±0.48
	GST–KLHL3^290–587^	FPPEPEEPEAD (WNK4 WT 554–564)	LB
1(F)	GST–KLHL3^290–587^	EPEEPEADQHQ (WNK4 WT 557–567	0.35±0.04
	GST–KLHL3^290–587^	ECEETEVDQHV (WNK3 WT 537–547)	0.84±0.05
	GST–KLHL3^290–587^	LQLDEETGEFL (NRF2 WT 74–84)	LB
	KEAP1 full-length	EPEEPEADQHQ (WNK4 WT 557–567	LB
	KEAP1 full-length	ECEETEVDQHV (WNK3 WT 537–547)	LB
	KEAP1 full-length	LQLDEETGEFL (NRF2 WT 74–84)	0.44±0.01
2(C)	GST–KLHL3^290–587^	EPEEPEADQHQ (WNK4 WT 557–567)	0.51±0.02
	GST–KLHL2^298–593^	EPEEPEADQHQ (WNK4 WT 557–567)	0.26±0.01

We went on to study the impact of truncating N- and C-terminal residues of the 19-residue WNK4 peptide to further characterize the WNK degron required for KLHL3 recognition. This revealed that removal of eight N-terminal residues (GPPSVFPP) had no appreciable effect on KLHL3 binding (Figure 1D and Table 1). However, removal of even just one C-terminal residue markedly reduced binding. All subsequent studies were therefore performed using the core 11-residue WNK4 peptide (EPEEPEADQHQ) that binds KLHL3 with high affinity (*K*_d_=0.69 μM). Interestingly, this sequence is identical in WNK1 and WNK4, whereas four of the 11 residues differ in WNK3 (Figure 1E). We therefore investigated whether these substitutions in WNK3 (E**C**EE**T**E**V**DQH**V**, where bold and underlined residues differ to WNK1/4 degron motifs) were compatible with high-affinity binding to KLHL3. We observed that despite these sequence differences, the WNK3 motif interacted with KLHL3 with similarly high affinity to WNK1/4 (Figure 1F and Table 1). We also found that the NRF2 degron motif bound strongly to KEAP1, but not to KLHL3 (Figure 1F and Table 1). Vice versa, the WNK1/4 degron failed to bind KEAP1 (Figure 1F and Table 1), confirming the selectivity of these substrate adaptor proteins.

### Structure determination of the WNK1/4 acidic degron motif bound to KLHL3 and KLHL2

**Figure 2:**
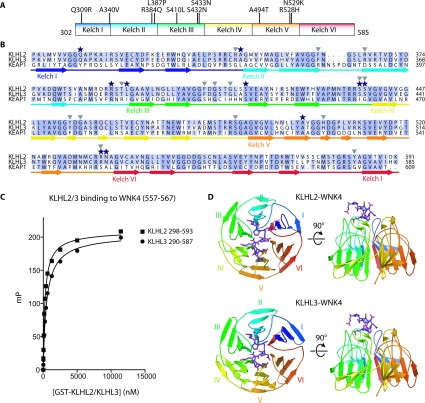
Overview of the KLHL2 and KLHL3 structures and their interaction with the WNK degron motif (**A**) Schematic representation of the Kelch domain (residues 302–585) of KLHL3 with the positions of the dominant Gordon's syndrome-associated mutations illustrated. (**B**) Sequence alignment of the Kelch domain of KLHL2, KLHL3 and KEAP1, with the secondary structure of KLHL3 indicated. A black star above the amino acid indicates the position of a Gordon's syndrome mutation and a grey triangle indicates key contact residues in the KLHL3–WNK4 structure. (**C**) Comparison of the binding of the WNK4 degron motif to KLHL2 and KLHL3. Fluorescent polarisation measurements were made as described in Figure 1 and the Materials and methods section. Purified GST–KLHL3^290–587^ or GST–KLHL2^298–593^ were mixed with the 11-residue WNK4 (557–567) degron peptide and binding curves were generated. (**D**) An overview of the KLHL2–WNK4 (top panel) and KLHL3–WNK4 (bottom panel) crystal structures. The Kelch domain is shown in cartoon representation and the WNK4 peptide is shown in purple stick representation.

The KLHL3 residues that are mutated in Gordon's syndrome are widely distributed across the Kelch domain of KLHL3 (Figure 2A). These residues are also strictly conserved in the highly homologous KLHL2 protein (Figure 2B) that has also been reported to bind WNK4 [25]. Consistent with this, we found that the WNK1/4 degron motif bound to the Kelch domain of KLHL2 with similar affinity to KLHL3 (Figure 2C). In contrast, most of the disease-causing mutations in KLHL3 are not conserved in the Kelch domain of KEAP1 (Figure 2B).

**Figure 3:**
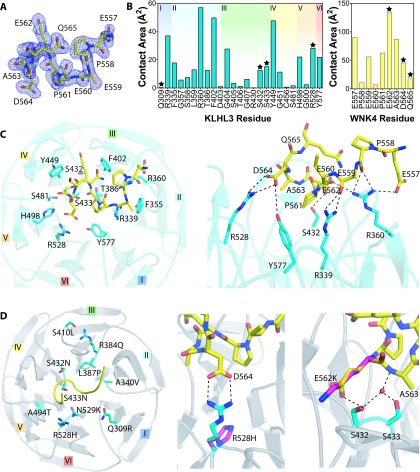
Interactions in the KLHL3–WNK4 complex (**A**) The conformation of the WNK4 11-mer peptide and corresponding electron density contoured at 1.0 σ for the KLHL3–WNK4 structure (PDB code 4CH9). (**B**) Buried surface area graphs for contact residues in the KLHL3–WNK4 complex. A black star indicates residues that are mutated in Gordon's syndrome. (**C**) An overview of the key WNK4-interacting residues (left-hand panel) and important polar contacts (right-hand panel) in the KLHL3–WNK4 interaction. KLHL3 is shown in light blue and WNK4 peptide in yellow. (**D**) The relative position of Gordon's syndrome-associated mutations found in KLHL3 (left-hand panel). Mutations R528H in KLHL3 (middle panel) and E562K in WNK4 (right-hand panel) affect residues with the highest buried surface area in each protein. The mutated residues are indicated in pink, with non-mutated residues in light blue (KLHL3) or yellow (WNK4). Dotted lines indicate hydrogen-bonding interactions. A bridging water molecule, between KLHL3 Ser^433^ and WNK4 Ala^562^ is indicated by a red sphere.

We determined the high-resolution crystal structures of the Kelch domains of human KLHL3 and KLHL2 in separate complexes with the 11-residue WNK4 degron motif (Figure 2D). Two Kelch domains were found in the asymmetric unit of each structure. The KLHL2 structure was solved at 1.6 Å (1 Å=0.1 nm) resolution in space group *P*4_3_2_1_2, whereas the structure of KLHL3 was determined at 1.8 Å in space group *P*2_1_2_1_2_1_. Data processing and refinement statistics for both structures are presented in Table 2. Electron density for the WNK4 peptide was clearly visible in both structures after initial molecular replacement and after refinement, the peptide was modelled into the binding site as shown in Figure 2(D) and Figure 3. For the KLHL2 structure, residues 292–305 from the N-terminus and residues 592–593 from the C-terminus were not clearly resolved in the electron density and were excluded from the model, as was the C-terminal residue of the WNK4 peptide (Gln^567^). Similarly, residues 296–299 and 586–587 of KLHL3 could not be modelled into the KLHL3–WNK4 complex, and WNK4 residues 566–567 and the side chains of Glu^559^ and Glu^560^ were also not defined (Figure 3A). The KLHL2 and KLHL3 structures were determined at pH 7.5 and pH 4.3 respectively, which probably contributes to differences in the resolution of particular residues. For example, protonation of WNK4 His^566^ may explain why this residue is not clearly resolved in the crystal structure of the KLHL3 complex. WNK4 Gln^567^ was disordered in both structures, consistent with a lack of stabilizing side-chain contacts, yet was critical for peptide binding (Figure 1D and Table 1). Although side-chain substitutions were tolerated at this position (for example, Val^547^ in WNK3), truncations were likely disfavoured by the introduction of an undesirable C-terminal carboxy group, or by changes induced in the conformation of the peptide backbone.

**Table 2:** Crystallographic data collection and refinement statistics Relevant crystallographic statistics for the structures of KLHL2 and KLHL3 with WNK4 peptide (PDB codes 4CHB and 4CH9 respectively). Values in parentheses indicates data for the highest resolution shell. ASU, asymmetric unit.

Parameter	KLHL2–WNK4 peptide	KLHL3–WNK4 peptide
Data collection		
Space group	*P*4_3_2_1_2	*P*2_1_2_1_2_1_
Cell constants		
*a*, *b*, *c* (Å)	117.7, 117.7, 106.65	84.76, 45.33, 146.79
α, β, γ (°)	90, 90, 90	90, 90, 90
Resolution (Å)	39.52–1.52 (1.57–1.52)	73.40–1.84 (1.90–1.84)
Unique observations	114962 (11326)	49110 (4342)
Completeness (%)	99.9 (39.52–1.56 Å)	98.0 (73.40–1.84 Å)
Redundancy	7.6 (7.0)	4.4 (3.0)
*R*_merge_	0.14 (1.1)	0.06 (0.25)
*I*/σ*I*	1.94 (at 1.52 Å)	3.58 (at 1.84 Å)
Refinement		
Resolution (Å)	39.52–1.56	73.40–1.84
MR model	2XN4	2XN4
Copies in ASU	2	2
*R*_work_/*R*_free_	0.177/0.194	0.144/0.184
Number of atoms	4998	5096
Average *B*-factor (Å^2^)	15.0	17.0
Average *B*-factor (protein) (Å^2^)	14.9	15.5
Average *B*-factor (peptides) (Å^2^)	27.3	33.9
Average *B*-factor (solvent) (Å^2^)	24.1	28.9
RMSD (bonds) (Å)	0.07	0.01
RMSD (angles) (°)	1.04	1.18
PDB code	4CHB	4CH9

The overall β-propeller structure of the Kelch domain from the BTB-Kelch family has been well described [17,35–38]. Six Kelch repeats, each composed of four antiparallel β-strands, form the six blades of the barrel structure. The final strand at the C-terminus pairs with the N-terminal strand to complete blade I (Figure 2D). In the present study, we show that KLHL3 and KLHL2 are structurally highly similar (Cα RMSD of 0.6 Å), and that both Kelch domains share a common mode of interaction with WNK4/1 peptide (Supplementary Figure S1A at http://www.biochemj.org/bj/460/bj4600237add.htm). Comparison of these structures with the KLHL2 apo (unbound) structure determined previously (PDB code 2XN4), reveals only minor conformational differences (Supplementary Figure S1B). This suggests that the KLHL3-binding site for WNK4 is essentially rigid and conserved across both proteins.

### Gordon's syndrome mutations affect key residues in the KLHL3–WNK4 interface

Owing to the high level of similarity between the KLHL2–WNK4 and KLHL3–WNK4 structures, all remaining discussion focuses on the KLHL3 structure. The binding interface between KLHL3 and WNK4 spans all six Kelch repeats and has a total buried surface area of 510 Å^2^, with blades II, III and IV participating in the greatest number of contacts (Figure 3B). Numerous contacts between KLHL3 and the WNK4 peptide can be observed, and together these interactions facilitate high-affinity binding between these two proteins. A number of KLHL3 residues provide significant hydrophobic contact with WNK4; including Phe^355^, Phe^402^, Gly^404^, Tyr^449^, Gly^451^ and His^498^ (Figure 3C). Among the KLHL3 residues, Arg^360^ contributes the greatest contact area and forms hydrogen bond interactions with WNK4 Glu^557^, as well as with the main chain atoms of WNK4 Pro^558^ and Glu^560^. KLHL3 Arg^339^ also forms a hydrogen bond with the main-chain carbonyl of WNK4 Glu^559^. Other important polar interactions involve residues that are mutated in some instances of Gordon's syndrome (Figure 3D). For example, WNK4 Glu^562^ has the greatest contact area of any interface residue and protrudes into the cleft between blades III and IV to hydrogen bond with KLHL3 Ser^432^ (Figure 3D, right-hand panel). Mutations have been described at both sites in patients with Gordon's syndrome (E562K and S432N respectively) [11,39] and would probably introduce steric clashes, as well the loss of hydrogen-bond formation. Perhaps the most notable interaction is the salt bridge formed between KLHL3 Arg^528^ and WNK4 Asp^564^ (Figure 3D, middle panel). Patients with Gordon's syndrome have been identified with mutations in either of these residues and notably mutation of Arg^528^ to a histidine residue is the most prevalent KLHL3 mutation described thus far. A histidine side chain at this position would potentially disrupt the local structure in KLHL3 and would be too short to maintain a hydrogen-bond interaction with WNK4. The WNK4 D564A mutation would similarly disrupt this interaction and indeed our independent binding experiments using either KLHL3 R528H or WNK4 D564A (Figures 1B and 1C) confirm the importance of this salt bridge for a high-affinity interaction. Finally, a buried water molecule is observed to mediate hydrogen bonding between WNK4 Ala^563^ and the side chains of KLHL3 Ser^432^ and Ser^433^ (Figure 3D, right-hand panel). Gordon's syndrome mutations at either position (S432N or S433N) would disrupt this interaction network.

Other Gordon's syndrome mutations in KLHL3, including Q309R and N529K, are not involved in direct contact between the two proteins, but are situated within close proximity to the peptide-binding site (Figure 3D). These substitutions most likely disrupt the interaction by unfavourably altering the shape and charge of the binding pocket and by perturbing neighbouring residues in the binding interface. For example, the N529K mutation is predicted to perturb both the salt bridge between KLHL3 Arg^528^ and WNK4 Asp^564^, and the van der Waals packing interactions of KLHL3 Tyr^577^. The importance of KLHL3 Asn^529^ for WNK4 binding is demonstrated by the decreased affinity of the WNK4 19-residue peptide for KLHL3 N529K relative to wild-type KLHL3 (Figure 1B).

The remaining mutations identified in the Kelch domain of KLHL3 (A340V, R384Q, L397P, S410L and A494T) lie in sites disparate from the binding area. Arg^384^ and Ser^410^ form an intramolecular hydrogen bond between blades II and III that would be lost upon mutation, whereas A340V, L387P and A494T affect residues buried in the wild-type structure. All of these mutations are predicted to have a negative impact on the overall tertiary structure and stability of the KLHL3 protein.

## DISCUSSION

The highlight of the present study is that it defines the binding between the acidic degron motif of the WNK kinases and KLHL3 and KLHL2. This provides a molecular mechanism for the capture of these kinases by the CRL3^KLHL3^ and CRL3^KLHL2^ ubiquitin ligase complexes. Furthermore, it offers a structural explanation for the disruptive effects of a number of Gordon's syndrome mutations that are described in WNK4 and the Kelch domain of KLHL3. These mutations would inhibit the ubiquitylation of WNK4, and probably other WNK isoforms, by the CRL3^KLHL3^ complex, and result in the cellular accumulation of these master regulators of blood pressure. This, in turn, would be predicted to lead to overactivation of SPAK/OSR1 and the NCC/NKCC2 ion co-transporters, and consequently, increased salt retention leading to hypertension.

Our data further support the recent finding that KLHL2 may also function as a second CUL3 adaptor for the WNK kinases [25]. The presented structures reveal that KLHL2 and KLHL3 bind to the WNK1/4 degron in an almost indistinguishable manner and maintain strict conservation of all critical contact residues. Thus far, no mutations in KLHL2 have been described in patients with Gordon's syndrome, but the binding data strongly suggest that KLHL2 will also play a role in regulating WNK isoforms. In future work, it would be interesting to explore the relative importance of KLHL3 and KLHL2 for regulation of WNK isoforms, which vary in their relative abundance within different tissues. KLHL2 is best known for its high expression in the brain [40], whereas KLHL3 may play a dominant role in the kidney, consistent with the effects of KLHL3 mutations in hypertension. Sequence comparisons across the BTB-Kelch family suggest that only KLHL3 and KLHL2 possess the conserved residues required to interact with the WNK degron motif [17].

To promote efficient ubiquitylation of NRF2 by the KEAP1–CUL3 ligase complex (CRL3^KEAP1^), both a high-affinity and a low-affinity binding site must be engaged [41]. Given our peptide-based approach, we did not investigate whether a second low-affinity binding site is required for WNK ubiquitylation. It would be interesting to investigate in future studies whether a second binding site does indeed exist in the WNK isoforms, and furthermore, whether all Kelch substrates may have a high- and low-affinity degron site to fine tune substrate capture. Alternatively, given that WNK isoforms are known to be able to form both hetero- and homo-dimers [42], these two high-affinity WNK degrons may be sufficient to engage separately the two Kelch domains within the KLHL3 dimer. In this model, WNK dimerization may also influence WNK ubiquitylation levels.

Our structural and biochemical data suggest that all WNK isoforms may be regulated by the CRL3^KLHL3^/CRL3^KLHL2^ ubiquitin–ligase complexes. We were surprised to find that, despite the WNK3 degron containing four changes out of 11 residues, it still bound with high affinity to KLHL3 (Figure 1F). The WNK2 degron contains only one residue change at the C-terminal position relative to the WNK4/1 degron (Figure 1E). Although we have not directly tested whether the WNK2 degron binds to KLHL3, this is likely, as this residue was disordered in the KLHL3 complex structure and therefore unlikely to make a significant contribution to binding. The current data therefore indicate that all WNK isoforms will bind to KLHL3 and KLHL2 with similar affinity, at least *in vitro*.

In future it will be important to explore how ubiquitylation of WNK isoforms by CRL3^KLHL3^ is regulated and whether differences in the degron motif sequence might have an impact on the regulation. Notably, there are no phosphorylatable residues within the WNK1/4 degron, whereas the WNK3 degron does contain a conserved threonine residue (Thr^541^ in humans). Phosphorylation of this threonine residue would be predicted to disrupt the peptide interactions observed in the crystal structures and therefore WNK3–KLHL3/2 interactions could be regulated by phosphorylation. In the case of the KEAP1 system, in non-redox stressed cells, CRL3^KEAP1^ binds to and ubiquitylates the transcription factor NRF2, targeting it for proteasomal degradation [43]. Under conditions of oxidative stress, cysteine modification of KEAP1 leads to structural changes that prevent binding to NRF2, thereby inhibiting NRF2 ubiquitylation resulting in its markedly increased expression [44]. By analogy, regulation of WNK ubiquitylation may be controlled at the level of KLHL2/KLHL3. For example, in future work it would be instructive to explore whether osmotic conditions, known to greatly influence the WNK signalling pathway, alter the ability of KLHL2/KLHL3 to bind to the WNK degron or, to ubiquitylate WNK isoforms.

**Figure 4:**
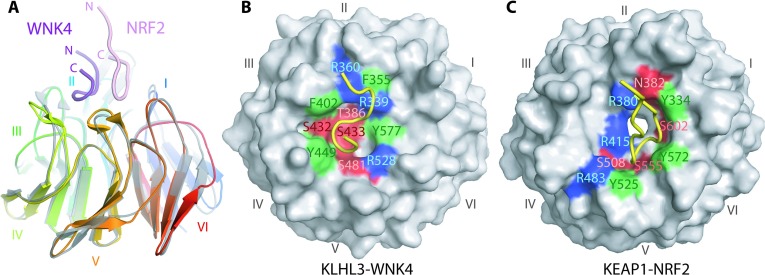
Comparison of WNK4 and NRF2 binding modes (**A**) The KLHL3–WNK4 structure (rainbow cartoon representation) was superimposed with that of the KEAP1–NRF2 complex (PDB code 2FLU) (grey). The WNK4 peptide (purple) is positioned in the opposite orientation and across different areas of the Kelch domain relative to the NRF2 peptide (pink). (**B** and **C**) Two corresponding surface representations of KLHL3–WNK4 and KEAP1–NRF2, with bound peptides shown as yellow ribbons, reveal markedly different binding pockets. Key contact residues are highlighted according to their properties; blue indicates a basic, red indicates polar and green indicates hydrophobic residues.

To date, structural studies of substrate recognition within the BTB-Kelch family have been limited to KEAP1, including complexes with NRF2 [37,38,45], the degron motifs of prothymosin-α [46] and sequestosome-1 [47]. Overall, these substrates were bound to KEAP1 in an almost identical position suggesting a preferred interaction site for Kelch–substrate interactions. Despite a similar acidic character, the WNK degron motif binds KLHL2/KLHL3 in a strikingly different manner to the KEAP1–substrate complexes (Figure 4A). Indeed, WNK4 and NRF2 peptides not only bind at different positions within the Kelch domain, but they are also orientated opposite to one another in terms of their N- and C-termini (Figure 4A). The NRF2 peptide makes greatest contact with blades I, IV and V of the KEAP1 Kelch domain, compared with blades II, III and IV for the KLHL2/3–WNK4 complex. Despite the apparent structural similarity of the Kelch domain backbones, the binding pockets bear no resemblance to one another in terms of both 3D shape and the position of contact residues across the pocket (Figures 4B and 4C). These differences help to explain the exquisite specificity of these adaptor proteins for their substrates, which is essential for ensuring the specific regulation of ubiquitylation, and also clearly have implications for the development of selective small-molecule inhibitors of the KEAP1 Kelch domain [48].

Mutations in KEAP1 have also been identified in human disease, most notably cancers of the lung, breast and gall bladder [37,49,50]. Parallel to Gordon's syndrome mutations in KLHL3, several of the reported KEAP1 mutations directly perturb the interface with NRF2, whereas others are likely to disrupt the structural integrity of the Kelch domain fold. Of these, many are found at the recurring double glycine motif on the second β-strand of Kelch repeats I, II, III and IV. The double glycine motif is conserved in KLHL2 and KLHL3 (Figure 2B), but there are currently no reported Gordon's syndrome mutations at these sites. Most described Gordon's syndrome mutations in KLHL3 are located either close to the peptide binding site or more randomly throughout the structure with no recognizable pattern between the Kelch repeats. It is clear that, despite the broad similarities in the structural fold of KEAP1 and KLHL2/KLHL3, there are crucial differences in the way these proteins interact with their substrates.

The findings described in the present paper provide important new insights into the regulation of WNK kinases by CRL3^KLHL3^ and, moreover, shed light on the very diverse mode of interactions between substrates and their E3 ligases. In the future, it will be important to determine the contribution of KLHL2 to blood pressure regulation, to understand if all WNK isoforms are indeed substrates of CRL3^KLHL3^ and to elucidate if and how WNK degradation is regulated by external signals.

## Online data

Supplementary data
