# Supplementary material for: Structural and biochemical characterization of the KLHL3–WNK kinase interaction important in blood pressure regulation
Source: Biochem J. 2014 May 13;460(Pt 2):237–46. doi: 10.1042/BJ20140153 (PMC4019986; doi:10.1042/BJ20140153)
Supplement: Supplementary data [file bj4600237add.pdf]

BJ Signal

Biochem. J. (2014) **460**, 237–246 (Printed in Great Britain) doi:10.1042/BJ20140153

online data

SUPPLEMENTARY ONLINE DATA

Structural and biochemical characterization of the KLHL3–WNK kinase interaction important in blood pressure regulation

Frances-Rose SCHUMACHER*[1], Fiona J. SORRELL†[1], Dario R. ALESSI*, Alex N. BULLOCK†[2] and Thimo KURZ*[2]

*MRC Protein Phosphorylation and Ubiquitylation Unit, College of Life Sciences, University of Dundee, Dow Street, Dundee DD1 5EH, Scotland, U.K.
†Structural Genomics Consortium, University of Oxford, Old Road Campus, Roosevelt Drive, Oxford OX3 7DQ, U.K.

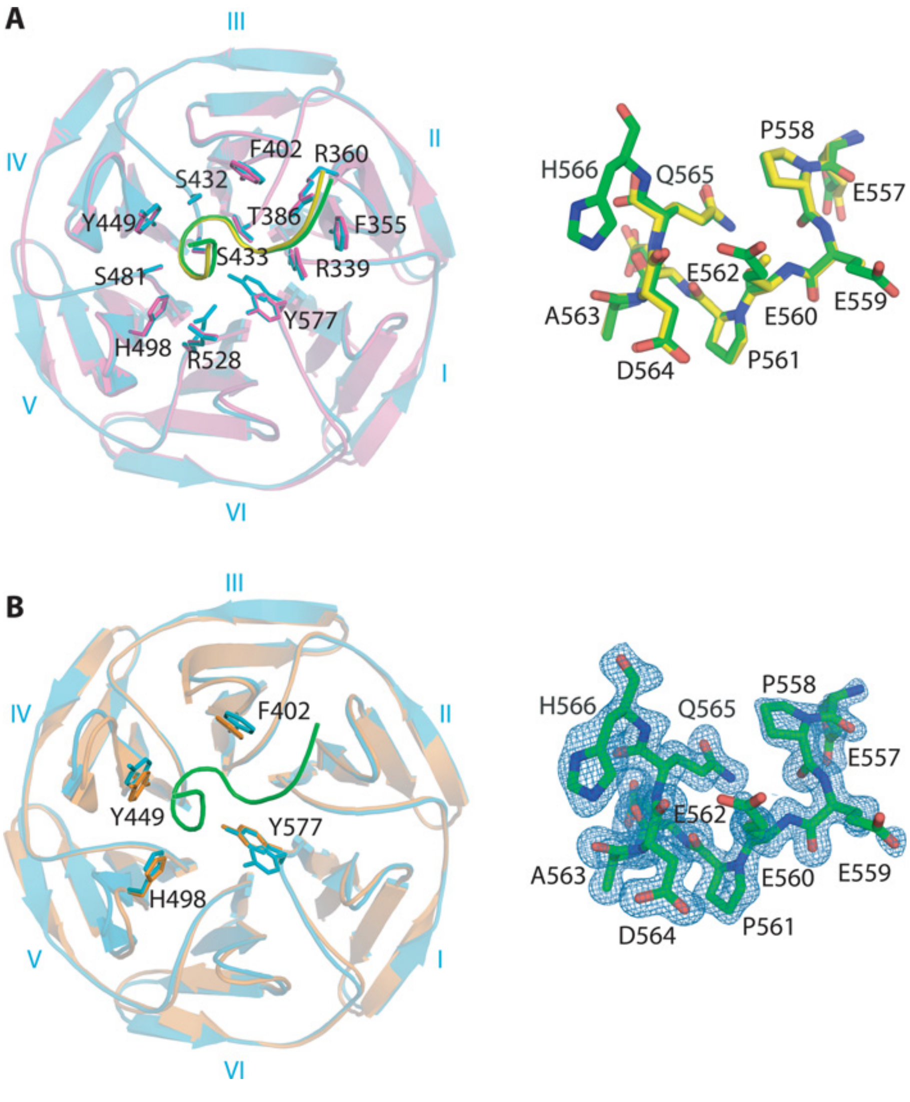

Figure S1 KLHL2–WNK4 peptide complex

(**A**) Superimposition of the crystal structures of KLHL2–WNK4 (blue with green peptide) and KLHL3–WNK4 (pink with yellow peptide) reveals the similarity between the two complexes. The key contact residues are labelled (left panel). Multiple conformations are visible for Arg^{528} and Tyr^{577} in the KLHL2 structure. His^{566} and the side chains from Glu^{559} and Glu^{560} of WNK4 are traceable in the KLHL2 structure, but are absent in the KLHL3 structure (right-hand panel). (**B**) Comparison of the apo (orange, PDB code 2XN4) and WNK4-bound structures of KLHL2 (blue) reveals the rigidity of the Kelch β-propeller and peptide-binding site. Electron density for the bound peptide is shown on the right-hand panel.

Received 3 February 2014/18 March 2014; accepted 18 March 2014
Published as BJ Immediate Publication 18 March 2014, doi:10.1042/BJ20140153

[1] These authors contributed equally to this work.
[2] Correspondence may be addressed to either of these authors (email alex.bullock@sgc.ox.ac.uk or t.kurz@dundee.ac.uk).
The structural co-ordinates reported for KLHL2 and KLHL3 will appear in the PDB under codes 4CHB and 4CH9 respectively.

© 2014 The Author(s)
The author(s) has paid for this article to be freely available under the terms of the Creative Commons Attribution Licence (CC-BY) (http://creativecommons.org/licenses/by/3.0/) which permits unrestricted use, distribution and reproduction in any medium, provided the original work is properly cited.

Biochemical Journal www.biochemj.org
